# Exploring the severity and risk factors of non-alcoholic fatty liver disease using the SAF scoring system

**DOI:** 10.3389/fmed.2025.1510679

**Published:** 2025-03-28

**Authors:** Xinxin Li, Shiyu Wang, Ziyu Zhang, Wen Deng, Yaqin Zhang, Weihua Cao, Xin Wei, Zixuan Gao, Linmei Yao, Shuojie Wang, Wei Yi, Yao Xie, Minghui Li

**Affiliations:** ^1^Department of Hepatology Division 2, Beijing Ditan Hospital, Capital Medical University, Beijing, China; ^2^Department of Gynecology and Obstetrics, Beijing Ditan Hospital, Capital Medical University, Beijing, China; ^3^HBV Infection, Clinical Cure and Immunology Joint Laboratory for Clinical Medicine, Capital Medical University, Beijing, China; ^4^Department of Hepatology Division, Peking University Ditan Teaching Hospital, Beijing, China

**Keywords:** non-alcoholic fatty liver disease (NAFLD), SAF scoring system, liver fibrosis, metabolic syndrome, risk factors, body mass index (BMI), uric acid, liver stiffness

## Abstract

**Objective:**

The steatosis, activity, and fibrosis (SAF) score is a histological scoring system developed by the European Association for the Study of the Liver to evaluate liver biopsy samples in cases of non-alcoholic fatty liver disease (NAFLD). Based on histopathological results and SAF scores, NAFLD patients were categorized into mild, moderate, and severe groups. We compared the differences between these groups and identified the risk factors influencing lesion severity.

**Methods:**

We gathered data from 539 NAFLD patients who underwent percutaneous liver biopsy confirmation at Beijing Ditan Hospital between January 2018 and December 2022. All biopsies were graded according to the SAF scoring system, and the severity of the disease was classified as mild, moderate, or severe. We compared the differences in gender, age, BMI, history of diabetes, history of hypertension, aspartate aminotransferase (AST), alanine aminotransferase (ALT), serum cholesterol levels, and other factors among NAFLD patients with varying degrees of disease severity. Additionally, we explored the risk factors that influenced the severity of lesions.

**Results:**

A total of 539 patients were enrolled in this study, with ages ranging from 6 to 79 years. Among them, there were 325 men and 214 women in an average age of 39 ± 13 years. The patients were divided into three groups based on disease severity: mild NAFLD group (162 cases), moderate NAFLD group (210 cases), and severe NAFLD group (167 cases). The results showed significant differences between the three groups in terms of age composition, high-calorie diet, family history of hypertension, ALT, AST, GGT, total bile acids, cholinesterase, glycosylated albumin, blood glucose, uric acid, type III procollagen, serum human laminin, liver stiffness, and hepatic steatosis.

**Conclusion:**

BMI, uric acid, AST, type III procollagen, liver stiffness, and hepatic steatosis play critical roles in the progression of NAFLD and contribute to high pathological SAF scores in NAFLD patients.

## 1 Introduction

In the 1980s, Ludwig et al. first reported on 20 patients with unexplained non-alcoholic fatty liver disease (NAFLD), whose liver biopsy specimens showed significant fat changes accompanied by lobular hepatitis (1). Since then, the concept of NAFLD has emerged (2). NAFLD is a common liver disease characterized by the presence of large vesicular steatosis in ≥5% of liver cells without a clear cause for the fat accumulation. It includes non-alcoholic fatty liver (NAFL), non-alcoholic steatohepatitis (NASH), liver fibrosis, cirrhosis, and liver cancer that may develop from NAFL (3). NAFLD is one of the most significant causes of liver disease worldwide, affecting approximately 24% of the global population, with an estimated prevalence of 27% in Asia (4). Although simple steatosis is typically regarded as a benign condition, its association to liver fibrosis can lead to cirrhosis and hepatocellular carcinoma. Over time, NAFLD may become a leading cause of end-stage liver disease (5).

The pathogenesis of NAFLD is not fully understood and involves complex interactions between multiple organs and signaling pathways. Key drivers include metabolic dysregulation, genetic predisposition, and lifestyle factors. Currently implicated mechanisms in NAFLD development include lipid accumulation (6), insulin resistance (7), oxidative stress (8), endoplasmic reticulum stress (9), and gut-liver axis dysregulation (10). NAFLD is intricately linked to metabolic abnormalities, with metabolic syndrome (MetS) representing one of the most significant risk factors for its development. MetS typically encompasses increased waist circumference, hyperglycemia, insulin resistance, dyslipidemia, and hypertension, all of which contribute to the histological progression of NAFLD. Previous studies have indicated that the prevalence of non-alcoholic steatohepatitis (NASH) and advanced fibrosis is notably higher in individuals with both diabetes and NAFLD compared to those with NAFLD alone (7). Hypertension is closely related to the progression of fibrosis in NAFLD. Longitudinal cohort studies involving NAFLD patients (11) have shown that hypertension increases the risk of fibrosis progression during follow-up in these individuals. A large cohort study in the United States revealed that the prevalence of NAFLD continues to rise, coinciding with increasing rates of diabetes, obesity, dyslipidemia, and hypertension. This highlights the significant metabolic interplay between these risk factors and NAFLD (12). These findings emphasize that metabolic abnormalities, such as diabetes and hypertension, are closely associated with the severity and progression of NAFLD, highlighting the critical role of metabolic syndrome in its pathogenesis. The leading causes of mortality in NAFLD patients are cardiovascular disease and non-liver malignancies. Studies have shown that NAFLD is closely associated with increased arterial stiffness, myocardial remodeling, kidney disease, and heart failure, followed by liver-related complications, the severity of liver fibrosis, and mortality rates (13). Therefore, to better reflect the disease process, experts proposed the concept of metabolic-associated fatty liver disease (MAFLD) in 2020, which refers to fatty liver diseases associated with systemic metabolic disorders (14). Furthermore, research has demonstrated that genetic predisposition plays a critical role in NAFLD susceptibility, with variants in genes such as MTARC1 being closely associated with an increased risk of NAFLD and its progression to NASH (15).

To further explore the risk factors influencing the severity of NAFLD, we collected pathological and clinical data from NAFLD patients diagnosed via liver histopathology. We compared the clinical indicators of NAFLD patients with varying degrees of liver disease severity using the SAF score and analyzed the risk factors influencing the severity of lesions in these patients. This analysis provides valuable insights for the clinical management and treatment of NAFLD patients.

## 2 Materials and methods

### 2.1 Research objects

**Figure 1:**
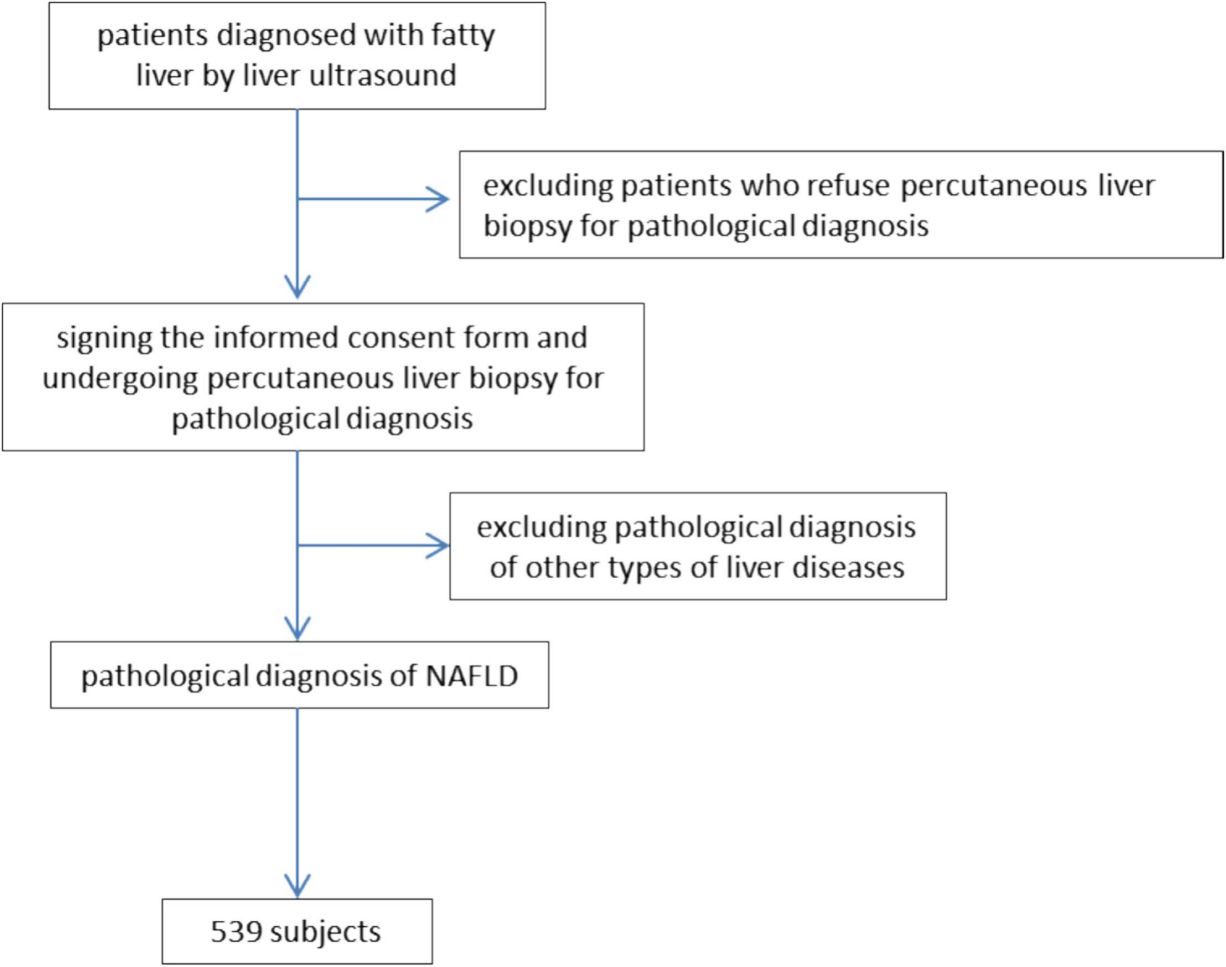
Patient enrollment flowchart.

NAFLD patients who underwent percutaneous liver biopsy at the Department of Hepatology Division 2, Beijing Ditan Hospital, affiliated to Capital Medical University, between January 2018 and December 2022 were included in this study. Clinical data, including gender, age, height, weight, sedentary lifestyle, high-calorie diet, history of hypertension, history of diabetes, and overall lifestyle, were collected. Additionally, clinical tests, including liver function, renal function, blood glucose levels, cholesterol, and triglycerides, and imaging tests such as liver ultrasound and transient elastography were performed. Information regarding liver histopathological diagnosis was also recorded, as illustrated in Figure 1.

#### 2.1.1 Inclusion and exclusion criteria

The inclusion and exclusion criteria are as follows: (1) Patients must be diagnosed with NAFLD through a pathological examination of liver tissue; (2) they should have complete clinical examination data; (3) they must have completed the questionnaire survey; (4) Individuals with alcoholic liver disease (defined as alcohol consumption>30 g/day for men and > 20 g/day for women) were excluded; and (5) Patients who had other liver diseases such as chronic viral hepatitis, autoimmune disorders, drug-induced liver injury, vascular issues, hereditary hemochromatosis, and Wilson’s disease were also excluded.

This study was approved by the Ethics Committee of Beijing Ditan Hospital, which is affiliated with Capital Medical University (Jingdilunke Zi [2018] No. (052-01)). All patients provided written informed consent before undergoing liver biopsy.

#### 2.1.2 Diagnostic criteria for liver tissue biopsy and histopathology

After the subjects signed the informed consent, a 16G liver puncture needle was used under ultrasound guidance to obtain liver tissue. The length of the tissue specimen collected had to be at least 1.0 cm (1.5–2.5 cm). Liver biopsy specimens were sliced consecutively and subjected to routine H&E staining, reticulin staining, and/or Masson staining. The Scheuer scoring system was used to evaluate the stages of liver fibrosis (S0–S4) and inflammation grading (G0–G4), with S3–S4 indicating advanced liver fibrosis. According to the Brunt grading system, fat degeneration was evaluated and divided into four levels: F0 (<5%), F1 (5% ~ 33%), F2 (33% ~ 66%), and F3 (≥ 66%). All pathological sections were independently observed and evaluated by two experienced pathologists. In the event of any disagreement, a third pathologist would serve as an arbitrator.

According to the histological severity of NAFLD, patients were divided into three groups using the SAF score (16, 17): mild NAFLD group, with SAF fibrosis and/or activity scores not exceeding one point; moderate NAFLD group, with SAF fibrosis and/or activity scores equal to two points; and severe NAFLD group, with SAF fibrosis and/or activity scores of three points or higher.

### 2.2 Test method

#### 2.2.1 Biochemical testing

All subjects were required to fast overnight before the blood draw, which involved an 8 h fast. This fasting was necessary to collect blood samples for routine testing of various parameters, including aspartate aminotransferase (AST), alanine aminotransferase (ALT), gamma-glutamyl transferase (GGT), bilirubin, total bile acids, cholesterol, creatinine, uric acid, fasting blood glucose, glycated hemoglobin, glycated albumin, C-peptide, insulin levels, homocysteine, alpha-fetoprotein (AFP), type III procollagen, and serum human laminin.

#### 2.2.2 Detection of liver transient elastography technology

All participants were required to fast for at least 3 h prior to the examination. During the procedure, patients were in a supine position with their right arm fully extended. The right hepatic lobe was scanned through the intercostal space to gather measurements. Utilizing ultrasound imaging, the operator placed the probe on a liver segment with a thickness of at least 6 cm, ensuring that it was free from large vascular structures and the gallbladder. The probe button was pressed to initiate the measurement, and 10 valid measurements were obtained. The median of these 10 measurements was recorded as the liver elasticity value. The liver fat attenuation parameter, expressed in dB/m, represents the average estimated value of ultrasound attenuation at a frequency of 3.5 MHz. The liver stiffness value, expressed in kPa, is the average estimate of Young’s modulus at a shear wave frequency of 50 Hz.

## 3 Definition of relevant indicators

### 3.1 Diabetes

Diabetes is defined as a condition based on the diagnostic criteria outlined in the 2020 version of the Guidelines for the Prevention and Treatment of Type 2 Diabetes in China, issued by the Diabetes Branch of the Chinese Medical Association (18, 19). It is characterized by a fasting blood glucose level of ≥7.0 mmol/L and includes patients who have previously been diagnosed with diabetes.

### 3.2 Hypertension

According to the 2018 revised version of the “Guidelines for the Prevention and Treatment of Hypertension in China,” released by the Hypertension Professional Committee of the Chinese Medical Association, patients with systolic blood pressure (SBP) of ≥140 mm Hg and/or diastolic blood pressure (DBP) of ≥90 mm Hg, as well as those previously diagnosed with hypertension, are defined as having hypertension (20).

### 3.3 Hyperlipidemia

According to the diagnostic criteria in the 2023 edition of the Chinese Blood Lipid Management Guidelines published by the Joint Expert Committee on the Revision of Chinese Blood Lipid Management Guidelines, hyperlipidemia is diagnosed when one or more of the following conditions are met: total cholesterol (TC) of 240 mg/dL (6.2 mmol/L) or higher, low-density lipoprotein cholesterol (LDL) of 160 mg/dL (4.1 mmol/L) or higher, high-density lipoprotein cholesterol (HDL) lower than 40 mg/dL (1.0 mmol/L), or triglycerides (TG) of 200 mg/dL (2.3 mmol/L) or higher (21).

### 3.4 BMI

The overweight and obesity limits recommended by the Chinese Obesity Working Group of the International Society for Life Sciences in 2002 are as follows: BMI < 18.5 kg/m2 indicates underweight, 18.5 kg/m2 ≤ BMI ≤ 23.9 kg/m2 indicates normal weight, 24.0 kg/m2 ≤ BMI ≤ 27.9 kg/m2 indicates overweight, and BMI ≥ 28.0 kg/m2 indicates obesity (22).

### 3.5 A sedentary lifestyle

A sedentary lifestyle refers to a person’s lack of sufficient physical activity in daily life, typically involving more than 8 h of sitting or lying down each day.

### 3.6 A high-calorie diet

A high-calorie diet typically refers to a dietary pattern in which the total energy intake (calories) exceeds an individual’s daily energy expenditure and basal metabolic needs.

## 4 Statistical method

All data were analyzed using IBM SPSS Statistics 25. Count data are expressed as percentages (%) using the chi-squared test. Measurement data that follow a normal distribution are expressed as means ± standard deviations. A *t*-test is conducted, and a one-way analysis of variance is performed for comparisons between multiple groups. Quantitative data that do not have a normal distribution are represented as quartiles, and independent sample non-parametric tests are performed. Pearson or Spearman correlation analyses were performed to identify factors related to the severity of NAFLD in patients. Regression analysis was performed to identify potential influencing factors, incorporating meaningful variables into logistic regression analysis to explore independent risk factors for the severity of NAFLD. A *p-*value of <0.05 indicates statistical significance.

## 5 Results

### 5.1 General demographic information of the research subjects

This study included a total of 539 research subjects, aged 6 to 79 years, comprising 325 men and 214 women who met the inclusion and exclusion criteria. There were 162 cases in the mild NAFLD group, with an average age of 41.39 ± 12.15 years, including 110 men (67.90%) and 52 women (32.10%). There were 210 cases in the moderate NAFLD group, with an average age of 39.84 ± 12.78 years, comprising 119 men (56.67%) and 91 women (43.33%). There were 167 cases in the severe NAFLD group, with an average age of 37.43 ± 14.12 years, including 96 men (57.49%) and 71 women (42.51%). According to non-parametric testing, there was a statistically significant difference in age between the three groups (*H = 7.250*, *p = 0.027*) but there was no statistically significant difference in gender (*χ*^2^ *= 5.621*, *p = 0.06*), as shown in Table 1. Subsequently, Dunn’s *t*-test was conducted to compare the ages between the three groups of patients pairwise, indicating a difference in age between the mild (median 41.5) and severe (median 35) groups (*p = 0.007*).

**Table 1:** General demographic data of study subjects.

Project	Total (*n* = 539)	Mild NAFLD group (*n* = 162)	Moderate NAFLD group (*n* = 210)	Severe NAFLD group (*n* = 167)	*H/χ^2^*	*p* value
Age (years)		41.39 ± 12.15	39.84 ± 12.78	37.43 ± 14.12	7.25	0.027
Gender (men, %)	325 (60)	110 (68)	119 (57)	96 (57)	5.621	0.06
High calorie diet (%)	415 (77)	95 (59)	173 (82)	147 (88)	45.713	<0.001
Sedentary lifestyle (%)	512 (95)	152 (94)	200 (95)	160 (96)	0.723	0.697

### 5.2 Comparison of dietary and lifestyle habits between the three groups

Comparing the distribution of dietary preferences and lifestyle habits, the results demonstrated a statistically significant difference in high-calorie diets between these groups (*p < 0.05*), as shown in Table 2. Furthermore, pairwise comparisons of high-calorie diets indicated significant differences between the mild and severe groups (*χ*^2^ *= 36.497*, *p < 0.001*), as well as between the mild and moderate groups (*χ2 = 25.588*, *p < 0.001*)*. P*atients in the severe group exhibited the highest proportion of high-calorie diets.

**Table 2:** History of metabolic diseases, family history, and BMI data in three groups.

Project	Total (*n* = 539)	Mild NAFLD group (*n* = 162)	Moderate NAFLD group (*n* = 210)	Severe NAFLD group (*n* = 167)	*χ^2^*	*p*
Diabetes (%)	51 (9)	11 (7)	25 (12)	15 (9)	2.858	0.24
Hypertension (%)	62 (12)	18 (11)	22 (10)	22 (13)	4.284	0.369
Hyperlipidemia (%)	275 (51)	71 (44)	113 (54)	91 (54)	4.813	0.09
Lipid-lowering drugs (%)	17 (3)	6 (4)	6 (3)	5 (3)	0.235	0.889
Family history of diabetes (%)	119 (22)	33 (20)	44 (21)	42 (25)	1.345	0.51
Family history of hypertension (%)	111 (21)	37 (23)	32 (12)	42 (25)	6.303	0.043
Family history of liver cancer (%)	24 (4)	7 (4)	11 (5)	6 (4)	0.601	0.74
Normal weight (%)	43 (8)	26 (16)	10 (5)	7 (4)	80.593	
Overweight (%)	197 (37)	92 (57)	65 (31)	40 (24)		
Obesity (%)	229 (55)	44 (27)	135 (62)	120 (72)	<0.001	

### 5.3 Comparison of metabolic diseases and BMI between the three groups

The distribution of BMI and related medical history (diabetes, hypertension, hyperlipidemia, and current use of lipid-lowering drugs), as well as family history (diabetes, hypertension, and liver cancer) among the three groups were compared. The results indicated no significant differences between the groups regarding diabetes, high blood pressure, hyperlipidemia, current use of lipid-lowering drugs, and family history of diabetes and liver cancer, as shown in Table 2. Compared to the mild group, the moderate NAFLD group exhibited a higher BMI (*H = 67.573, p < 0.001*) and a higher obesity rate (62% vs. 27%), while the severe NAFLD group also showed a higher BMI and a higher obesity rate (*H = 88.922*, *p < 0.001*). There was no significant difference in BMI between the moderate and severe groups (*H = 3.402*, *p = 0.065*). Our findings also suggested that the proportion of patients with a family history of hypertension in the severe group was higher than that in the moderate group (*χ*^2^ *= 5.793*, *p = 0.016*).

### 5.4 Comparison of biochemical indicators between the three groups

The results indicated statistically significant differences in SLT, AST, GGT, total bile acids, cholinesterase, glycosylated albumin, blood glucose, and uric acid between the three groups (*p < 0.05*). Additionally, the differences in type III procollagen, serum human laminin, liver stiffness, and liver steatosis—reflected as indicators of liver fibrosis—were also statistically significant among these groups. Dunn’s *t*-test pairwise comparisons revealed that the levels of alanine aminotransferase (118 > 90 > 55.5) and aspartate aminotransferase (59 > 51 > 30) in the severe and moderate groups exceeded those in the mild group, with patients in the severe group exhibiting the highest transaminase levels. Significant differences were observed in cholinesterase, total bile acids, glutamyl transpeptidase, blood glucose, and uric acid between the severe and mild groups, while no significant difference was found in glycosylated albumin between the moderate and severe groups. The degree of liver stiffness (9.35 > 7.4 > 6.2) and hepatic steatosis (285 > 267.5 > 217.5) in the severe group was more pronounced than in the moderate and mild groups. No significant difference was observed in type III procollagen and serum laminin between the moderate and severe groups. The three groups showed consistency in glycated hemoglobin, insulin levels, C-peptide, total bilirubin, alpha-fetoprotein, ApoA1, homocysteine, direct bilirubin, lipoprotein, ApoB, LDL, HDL, triglycerides, cholesterol, urea nitrogen, albumin, creatinine, and alkaline phosphatase, as shown in Tables 3, 4.

**Table 3:** Comparison of laboratory indicators between the three groups.

Project	Mild NAFLD group (*n* = 162)	Moderate NAFLD group (*n* = 210)	Severe NAFLD group (*n* = 167)	*H*	*p*
ALT (U/L)	55.5 (29.8, 89.8)	90 (55.8, 146)	118 (69, 164)	59.823	<0.001
AST (U/L)	30 (23, 45.3)	51 (30, 79)	59 (41, 96)	76.232	<0.001
GGT (U/L)	61 (29.8, 122.3)	68 (37.8, 112)	77 (49, 123)	6.568	0.037
Alkaline phosphatase	77 (65, 97.3)	81 (67, 107.3)	81.7 (66, 107)	2.461	0.292
Cholinesterase (U/L)	9272.5 (7,952, 10875.8)	9,774 (8378.8, 11138.3)	9,849 (8,548, 11,225)	6.023	0.049
Total bilirubin (umol/L)	12 (9.9, 17)	13 (9.7, 17)	12 (9.5, 16)	1.689	0.43
Direct bilirubin (umol/L)	3.95 (2.9, 6)	4.3 (3, 5.7)	3.900 (3.1, 5.4)	1.243	0.537
Total bile acid (umol/L)	4.3 (2.7, 6.8)	4.5 (2.8, 8.3)	5.3 (3.1, 9.3)	6.525	0.038
Cholesterol (mmol/L)	4.7 (4.1, 5.4)	4.78 (4.2, 5.5)	4.885 (4.1, 5.6)	1.439	0.487
Triglycerides (mmol/L)	1.725 (1.2, 2.3)	1.76 (1.3, 2.6)	1.695 (1.2, 2.5)	0.849	0.654
HDL (mmol/L)	1.03 (0.9, 1.2)	1.03 (0.9, 1.2)	1.04 (0.9, 1.2)	0.257	0.879
LDL (mmol/L)	2.73 (2.2, 3.2)	2.86 (2.4, 3.4)	2.985 (2.2, 3.4)	4.257	0.119
ApoA1 (g/L)	1.305 (1.1, 1.5)	1.32 (1.2, 1.5)	1.34 (1.2, 1.5)	2.565	0.277
ApoB (g/L)	0.875 (0.7, 1.1)	0.89 (0.7, 1.1)	0.91 (0.7, 1.1)	2.12	0.346
Lipoprotein (mg/dl)	7.45 (3.8, 14)	6.8 (3.1, 15.4)	7.05 (3.8, 21.6)	1.42	0.492
Albumin (g/L)	46 (44, 48)	46 (43, 49)	47 (43, 48)	0.232	0.891
Blood glucose (mmol/L)	5.63 (5.2, 6.2)	5.84 (5.3, 6.6)	5.775 (5.4, 6.8)	6.224	0.045
Glycated hemoglobin (%)	5.3 (4.8, 5.8)	5.4 (4.8, 6.2)	5.5 (4.9, 6.5)	5.583	0.061
Glycosylated albumin (%)	12.4 (10.4, 13.9)	12.8 (11.6, 15.3)	12.835 (11.4, 15.9)	8.595	0.014
Insulin levels (mU/L)	11.7 (6.2, 17.4)	13.03 (6.8, 18.3)	13.855 (7.6, 18.6)	5.127	0.077
C-peptide (ng/mL)	3.6 (2.9, 4.6)	3.6 (2.9, 4.9)	3.865 (3.2, 5.2)	4.393	0.111
Urea nitrogen (mmol/L)	4.79 (4, 5.5)	4.645 (4.1, 5.5)	4.545 (3.7, 5.5)	2.823	0.244
Creatinine (umol/L)	66 (57, 76)	63 (53.8, 75)	63 (52, 72.3)	5.813	0.055
Uric acid (umol/L)	339 (292, 412.5)	366 (282.5, 452.3)	386 (315, 464)	11.457	0.003
Homocysteine (umol/L)	12.6 (9.9, 16)	12 (8.9, 15.7)	12.245 (9.1, 16.8)	0.425	0.808
AFP (ng/ml)	3.2 (2.2, 4.8)	3.1 (2.2, 5.1)	3.1 (2.4, 5.8)	0.842	0.656
Type III procollagen (ug/L)	15.315 (9.8, 23)	18.35 (12, 35.1)	17.42 (12.8, 41.4)	11.48	0.003
Serum human laminin (ug/L)	76.36 (60.4, 106.3)	92.245 (71.8, 112.9)	86.16 (70.5, 112.6)	8.871	0.012
Liver stiffness (kPa)	6.2 (5.3, 7.5)	7.4 (5.9, 10.1)	9.35 (6.8, 15.6)	76.501	<0.001
Liver steatosis (dB/m)	217.5 (180, 276.8)	267.5 (245, 290.5)	285 (255.8, 309.3)	65.524	<0.001

The laboratory indicator data above are all non-normally distributed quantitative data, expressed as M (Q1, Q3).

**Table 4:** Pairwise comparison results using Dunn’s test.

	(I)name	(J)name	(I1)median	(J1)median	*I1-J1*	*p*
ALT (U/L)	Mild	Moderate	55.5	90	−34.5	0.000
	Mild	Severe	55.5	118	−62.5	0.000
	Moderate	Severe	90	118	−28	0.003
AST (U/L)	Mild	Moderate	30	51	−21	0.000
	Mild	Severe	30	59	−29	0.000
	Moderate	Severe	51	59	−8	0.002
GGT (U/L)	Mild	Moderate	61	68	−7	0.252
	Mild	Severe	61	77	−16	0.011
	Moderate	Severe	68	77	−9	0.12
Cholinesterase (U/L)	Mild	Moderate	9272.5	9,774	−501.5	0.068
	Mild	Severe	9272.5	9,849	−576.5	0.018
	Moderate	Severe	9,774	9,849	−75	0.503
Total bile acid (umol/L)	Mild	Moderate	4.3	4.5	−0.2	0.487
	Mild	Severe	4.3	5.3	−1	0.014
	Moderate	Severe	4.5	5.3	−0.8	0.057
Glycosylated albumin (%)	Mild	Moderate	12.4	12.8	−0.4	0.012
	Mild	Severe	12.4	12.835	−0.435	0.008
	Moderate	Severe	12.8	12.835	−0.035	0.781
Blood glucose (mmol/L)	Mild	Moderate	5.63	5.84	−0.21	0.052
	Mild	Severe	5.63	5.775	−0.145	0.018
	Moderate	Severe	5.84	5.775	0.065	0.575
Uric acid (umol/L)	Mild	Moderate	339	366.5	−27.5	0.076
	Mild	Severe	339	386.5	−47.5	0.001
	Moderate	Severe	366.5	386.5	−20	0.07
Type III procollagen (ug/L)	Mild	Moderate	15.315	18.35	−3.035	0.015
	Mild	Severe	15.315	17.42	−2.105	0.001
	Moderate	Severe	18.35	17.42	0.93	0.284
Serum human laminin (ug/L)	Mild	Moderate	76.36	92.245	−15.885	0.005
	Mild	Severe	76.36	86.16	−9.8	0.020
	Moderate	Severe	92.245	86.16	6.085	0.693
Liver stiffness (kPa)	Mild	Moderate	6.2	7.4	−1.2	0.000
	Mild	Severe	6.2	9.35	−3.15	0.000
	Moderate	Severe	7.4	9.35	−1.95	0.000
Liver steatosis (dB/m)	Mild	Moderate	217.5	267.5	−50	0.000
	Mild	Severe	217.5	285	−67.5	0.000
	Moderate	Severe	267.5	285	−17.5	0.008
	Moderate	Severe	267.5	285	−17.5	0.008

## 6 Correlation analysis of factors affecting the severity of NAFLD

To further investigate the factors affecting the SAF grading of NAFLD patients, we conducted a correlation analysis between various independent variables and the dependent variable (SAF grading). First, indicators reflecting liver fibrosis—type III procollagen, liver stiffness, and hepatic steatosis—were used as independent variables in a stepwise regression analysis. The resulting model was SAF = 0.705 + 0.005 * type III procollagen +0.028 * liver stiffness +0.004 * hepatic steatosis (*F = 31.909, p < 0.05*). The model’s *R*^2^ was 0.174, indicating that it explains 17.4% of the variation in SAF. The analysis revealed that type III procollagen (95% CI: 0.002–0.009), liver stiffness (95% CI: 0.018–0.037), and hepatic steatosis (95% CI: 0.002–0.005) positively influenced SAF grading. Subsequently, we performed a stepwise regression analysis using indicators of liver inflammation (alanine aminotransferase and aspartate aminotransferase) along with the remaining variables as independent predictors. The model was SAF = −0.844−0.001 * glutamyl transpeptidase +0.003 * aspartate aminotransferase +0.001 * uric acid − 0.009 * creatinine − 0.000 * urea nitrogen +0.103 * BMI, with an *R*^2^ of 0.256 (*F = 26.100, p < 0.05*). The results demonstrated that uric acid, aspartate aminotransferase, and BMI had a significantly positive impact on SAF, while glutamyl transpeptidase, creatinine, and urea nitrogen exhibited a significantly negative influence.

## 7 Discussion

In this study, data were gathered from 539 NAFLD patients, revealing differences in demographic characteristics, dietary and lifestyle habits, metabolic diseases, family history, and laboratory indicators among patients with varying degrees of severity. The key factors affecting the severity of NAFLD were also explored. This study showed that BMI, uric acid, AST, type III procollagen, liver stiffness, and hepatic steatosis indicators play important roles in the progression of NAFLD, suggesting that clinical monitoring and management of these indicators should be enhanced to improve the prognosis for NAFLD patients.

Our results showed that there were significant differences in the age composition of patients with mild, moderate, and severe NAFLD (*p = 0.027*), suggesting that age may be an important factor influencing the severity of NAFLD. As NAFLD severity increases, the average age of patients showed a decreasing trend, which contradicts the findings of previous research on the relationship between NAFLD and age (22, 23). In this study, the severe NAFLD group included eight underage patients (aged <18 years) with a BMI of ≥28 kg/m^2^ and liver biopsy results that met the criteria for severe NAFLD. While previous research has predominantly focused on adult populations, recent data indicate a significant rise in NAFLD prevalence among adolescents, closely associated with metabolic syndrome (24, 25). To validate the robustness of our findings, we conducted sensitivity analyses by repeating the regression models after excluding the underage patients. The results showed that the effect sizes of key risk factors, such as BMI, uric acid, and AST, changed by less than 5%, suggesting that the inclusion of underage patients did not significantly impact our findings. A study focused on adolescents also showed that the incidence and severity of NAFLD were notable among young people (26).

After excluding the underage patients, the impact of BMI on SAF scores decreased from *β* = 0.103 to 0.098 (*Δ* = 4.9%), and uric acid decreased from β = 0.001 to 0.0009 (Δ = 10%). These changes did not reach statistical significance (all *p > 0.05*). This study is the first to delineate the clinical characteristics of adolescent NAFLD patients within the SAF scoring framework, revealing that their high-risk metabolic phenotypes (e.g., severe obesity and hyperuricemia) align with those observed in adult populations. Although the limited sample size may constrain the power of subgroup analyses, sensitivity analyses support the robustness of the primary findings (Table 5). Given that young patients may be more inclined toward high-calorie diets and more susceptible to insulin resistance and metabolic syndrome, this suggests they may be more likely to develop severe NAFLD. This study identified key factors influencing the severity of NAFLD, including BMI, uric acid, AST, liver fibrosis markers, and hepatic steatosis. Notably, the gender distribution among severity groups approached statistical significance (χ^2^ = 5.621, *p* = 0.06). Although this result did not meet the conventional significance threshold, its potential clinical and biological implications warrant further investigation. The observed gender trend aligns with recent evidence suggesting gender-specific pathways in NAFLD progression. Men are more susceptible to visceral adiposity and insulin resistance, which may accelerate hepatic lipid accumulation and fibrosis (27), while premenopausal women may benefit from estrogen-mediated lipid metabolism regulation and an anti-inflammatory effect (28, 29). However, including adolescent patients in this study complicates this interpretation, as their hormonal environment differs from that of adults. To assess the robustness of the gender trend, we conducted sensitivity analyses by excluding underage patients (*n* = 8) and adjusting for age and BMI. After exclusion, the gender difference remained non-significant (χ^2^ = 4.872, *p* = 0.09), indicating that the trend was not driven by the pediatric subgroup. However, when stratified by BMI categories (normal, overweight, obese), obese men exhibited a higher proportion of severe NAFLD compared to women (65% vs. 48%, *p* = 0.08), suggesting that gender differences may interact with metabolic risk factors. The effect size for gender differences, calculated using Cramer’s V (V = 0.07), indicated a weak association. Although statistically modest, this finding is consistent with population-based studies reporting a 10–15% higher NAFLD prevalence in men compared to women. The clinical relevance of this trend lies in its potential to inform personalized screening strategies, particularly for obese men who may benefit from earlier intervention. These findings underscore the importance of considering gender-specific risk profiles in NAFLD management and highlight the need for further research to elucidate the underlying mechanisms.

**Table 5:** Sensitivity analysis results of regression models before and after excluding underage patients.

Variables	Total sample (*n* = 539)		After excluding underage patients (*n* = 531)		Δ%	Change in *p*-value
	*β* (95% CI)	*p*	*β* (95% CI)	*p*		
BMI (kg/m^2^)	0.103 (0.078–0.128)	<0.001	0.098 (0.073–0.123)	<0.001	−4.90%	0.32
Uric acid (μmol/L)	0.001 (0.0005–0.0015)	0.003	0.0009 (0.0004–0.0014)	0.005	−10.00%	0.45
AST (U/L)	0.003 (0.002–0.004)	<0.001	0.003 (0.002–0.004)	<0.001	0%	0.89
Liver stiffness (kPa)	0.028 (0.022–0.034)	<0.001	0.027 (0.021–0.033)	<0.001	−3.60%	0.51
Liver steatosis (dB/m)	0.004 (0.003–0.005)	<0.001	0.004 (0.003–0.005)	<0.001	0%	0.95

A multiple linear regression model was employed, with the SAF score as the dependent variable and adjusted variables including age, gender, diabetes history, and history of hypertension. Δ%: Percentage change in regression coefficients after excluding underage patients, calculated as (β_excluded − β_full) /β_full × 100%. *p*-value change: The significance of differences in *p*-values before and after exclusion was tested using the bootstrap method with 1,000 resampling iterations. Data format: *β* values are standardized regression coefficients, 95% CI represents the confidence interval, *p*-values are retained to three decimal places, and values less than 0.001 are labeled as extremely significant.

The results of the three groups indicated that patients in the moderate to severe groups were more inclined toward a high-calorie diet. The structure of this high-calorie diet, rich in saturated fatty acids and fructose, closely related to obesity, may lead to an energy surplus and fat accumulation, which could be one of the key mechanisms in the development of NAFLD. This aligns with the opinions in the “Diagnosis and Treatment Guidelines for Non-Alcoholic Fatty Liver Disease,” formulated by the Fatty Liver and Alcoholic Liver Disease Group of the Hepatology Branch of the Chinese Medical Association in 2018 (30). Clinical attention should focus on the dietary management of NAFLD patients, and early intervention in their diet and lifestyle habits should be implemented to prevent further disease progression. A sedentary lifestyle is also a risk factor for NAFLD; however, our research did not find a significant correlation with the SAF grading of NAFLD. We consider that a sedentary lifestyle does not directly contribute to MetS, such as hypertension, hyperglycemia, and obesity.

Our results also showed that the moderate and severe NAFLD groups had higher BMI and obesity rates compared to the mild group, while the severe group had a greater proportion of patients with a family history of hypertension than the moderate group.

Among the three groups, our research indicated that the ALT and AST levels in the severe and moderate groups were higher than those in the mild group, with the highest transaminase levels observed in the severe group. Additionally, levels of cholinesterase, total bile acids, GGT, blood glucose, uric acid, liver stiffness, and hepatic steatosis in the severe group were higher than those in the mild group, consistent with the progression of NAFLD. However, there were no significant differences in glycated hemoglobin, insulin levels, C-peptide, total bilirubin, alpha-fetoprotein, ApoA1, homocysteine, direct bilirubin, lipoprotein, ApoB, LDL, HDL, triglycerides, cholesterol, urea nitrogen, albumin, creatinine, and alkaline phosphatase among the three patient groups. This may be due to patients potentially having taken hypoglycemic or lipid-lowering medications in the past or recently altering their lifestyle habits through diet and exercise, which could affect their blood sugar and lipid levels. We only accounted for the current medication history and did not consider previous medication history or details related to blood glucose and lipid levels. Furthermore, some patients may not have monitored their blood glucose and lipid levels or may not have taken them seriously, leaving it unclear whether they had abnormal levels. Due to these interfering factors and the lack of important information, we believe that this part of the analysis does not accurately reflect the true results.

To further explore the factors affecting SAF grading in NAFLD patients, we conducted a correlation analysis between these factors and SAF grading. Stepwise regression analysis revealed that the fibrosis-related model (*R*^2^ = 0.174, *p* < 0.001) and the inflammation-metabolism model (*R*^2^ = 0.256, *p* < 0.001) explained 17.4 and 25.6% of the variation in SAF scores, respectively. For every 1 kg/m^2^ increase in BMI, the SAF score increased by 0.103 units (95% CI: 0.078–0.128), consistent with the 2023 American Association for the Study of Liver Diseases guidelines, which identify obesity as a key driver of NAFLD progression (3). Elevated uric acid levels promote hepatic steatosis by activating the NLRP3 inflammasome (31), while AST, reflecting mitochondrial dysfunction and hepatocyte injury, showed a positive association with SAF (*β* = 0.003), supporting its role as a marker of disease activity.

The results showed that BMI, uric acid, AST, type III procollagen, liver stiffness, and hepatic steatosis exhibited a positive impact on SAF grading, while GGT, creatinine, and urea nitrogen had a significant negative impact on SAF grading. Research suggests a correlation between high levels of uric acid and lipid accumulation or steatosis in liver cells. Elevated uric acid levels may increase oxidative stress and activate pro-inflammatory and pro-fibrotic cytokines, leading to fat accumulation, cellular damage, and inflammation in the liver (32, 33). Interestingly, GGT showed a negative correlation with the pathological SAF score in NAFLD, which appears counterintuitive, given its role as a marker of liver injury (34). We hypothesize that this may be due to several factors: the study sample, which included one-third of mild NAFLD patients, exhibited higher GGT levels but lower SAF scores, potentially introducing selection bias; GGT levels might be affected by uncontrolled factors such as medications and lifestyle choices, which were not adequately controlled in this study; and the liver may react to early fatty degeneration and inflammation through compensatory mechanisms, leading to elevated GGT levels that could decrease as the disease progresses to more severe stages (35).

Patients with NAFLD typically do not exhibit cholestasis; however, insulin resistance significantly enhances lipid mobilization in adipose tissue and the uptake and synthesis of triglycerides in hepatocytes, leading to lipid metabolism disorders and lipid peroxidation. Glutathione (GSH), the primary cellular thiol antioxidant, plays a crucial role in mitigating oxidative stress, while GGT is integral to GSH metabolism. Elevated GGT levels promote the hydrolysis of GSH to cysteinylglycine, which can be oxidized to generate reactive oxygen species (ROS), further triggering hepatic inflammation (36). This mechanism explains the increase in GGT levels during the early stages of NAFLD (F0–F1), which is associated with enhanced oxidative stress. However, as the disease progresses to advanced fibrosis (F3–F4), the reduction in hepatocyte number and function—along with miR-122 overexpression targeting GGT1 mRNA to inhibit its translation—may lead to decreased GGT release (37). Thus, the dynamic changes in GGT not only reflect the oxidative stress status in NAFLD but also serve as potential biomarkers for disease progression.

A study on obese Chinese people suggests that the GGT/HDL-c ratio may be associated with NAFLD (38). In recent years, numerous studies have explored the connection between the GGT/HDL-C ratio and NAFLD, demonstrating that this ratio is significantly correlated with the occurrence of NAFLD and exhibits superior diagnostic performance compared to GGT or HDL-C alone. However, research on the underlying mechanisms remains limited. It is hypothesized that elevated GGT levels indicate increased oxidative stress and hepatic inflammation, while decreased HDL-C levels are associated with lipid metabolism disorders and an elevated risk of atherosclerosis. The GGT/HDL-C ratio amalgamates these two pathophysiological processes, potentially providing a more comprehensive biomarker for NAFLD (39–41). In clinical practice, monitoring GGT levels may aid in assessing the degree of oxidative stress and the risk of disease progression in NAFLD patients, particularly when liver biopsy is not feasible. Future research should explore the combined application of GGT with other non-invasive markers (e.g., FIB-4, NFS) to improve the accuracy of NAFLD diagnosis and staging.

This study confirms the central role of BMI, uric acid, and AST in the progression of NAFLD, providing potential targets for individualized treatment. BMI, a core metabolic risk factor, has been shown to correlate with hepatic fat deposition and fibrosis. The 2023 AASLD guidelines emphasize that a 5–10% weight reduction can significantly improve hepatic steatosis and inflammation and even reverse early fibrosis (3). The positive effect of BMI on SAF scores (*β* = 0.103) in this study further underscores the central role of weight management in NAFLD treatment.

The association between hyperuricemia and NAFLD severity may be mediated by NLRP3 inflammasome activation. Recent animal studies suggest that uric acid promotes hepatic lipid accumulation through the ROS/JNK pathway (33). In this study, uric acid levels were significantly elevated in the severe group, highlighting the need for clinical attention to uric acid regulation, particularly in patients with comorbid metabolic syndrome. In clinical practice, a multidisciplinary approach is recommended for comprehensive metabolic risk management, combined with dynamic assessment of disease progression using non-invasive markers such as liver stiffness and the GGT/HDL-C ratio.

This study also has a few limitations. First, it is a single-center retrospective study with a limited sample size, which may result in selection bias. Second, it failed to comprehensively evaluate all factors that may affect the severity of NAFLD, such as genetic and environmental factors. Differences in alcohol and food consumption, physical activity, and medication use could not be statistically accounted for, and these factors cannot be dismissed as potential influences on the observed changes in fatty liver across groups. Finally, the use of questionnaires to assess participants’ medical histories of hypertension, hyperlipidemia, or diabetes failed to capture details such as disease duration, medications taken, and disease control. Future research should aim to expand the sample size and incorporate multicenter data to enhance the generalizability and reliability of the findings.

## Data Availability

The raw data supporting the conclusions of this article will be made available by the authors, without undue reservation.
